# BioSAXS at European Synchrotron Radiation Facility – Extremely Brilliant Source: BM29 with an upgraded source, detector, robot, sample environment, data collection and analysis software

**DOI:** 10.1107/S1600577522011286

**Published:** 2023-01-01

**Authors:** Mark D. Tully, Jerome Kieffer, Martha E. Brennich, Raphael Cohen Aberdam, Jean Baptiste Florial, Stephanie Hutin, Markus Oscarsson, Antonia Beteva, Anton Popov, Dihia Moussaoui, Pascal Theveneau, Gergely Papp, Jonathan Gigmes, Florent Cipriani, Andrew McCarthy, Chloe Zubieta, Christoph Mueller-Dieckmann, Gordon Leonard, Petra Pernot

**Affiliations:** a ESRF – The European Synchrotron, 71 Avenue des Martyrs, 38043 Grenoble, France; b EMBL Grenoble, 71 Avenue des Martyrs, 38042 Grenoble, France; c LPCV, CNRS, 17 Avenue des Martyrs, 38054 Grenoble, France; University of Essex, United Kingdom

**Keywords:** BM29, high-throughput small-angle X-ray scattering, SAXS, macromolecules in solution, online size-exclusion chromatography (SEC-SAXS), microfluidic screening

## Abstract

As part of its Extremely Brilliant Source (EBS) upgrade project, the ESRF’s BM29 BioSAXS beamline was subject to a significant upgrade and refurbishment. In addition to the replacement of the beamline’s original bending magnet source by a two-pole wiggler, leading to an increase in brilliance by a factor of 60, the sample environment of the beamline was almost completely refurbished: a vacuum-compatible Pilatus3 X 2M with a sensitive area of 253.7 mm × 288 mm and frame rates up to 250 Hz was installed, increasing the active area available and thus the *q*-scaling of scattering images taken; the sample changer was replaced with an upgraded version, allowing more space for customizable sample environments and the installation of two new sample exposure units; the software associated with the beamline was also renewed. In addition, the layout and functionality of the *BSXCuBE3* (*BioSAXS Customized Beamline Environment*) data acquisition software was redesigned, providing an intuitive ‘user first’ approach for inexperienced users, while at the same time maintaining more powerful options for experienced users and beamline staff. Additional features of *BSXCuBE3* are queuing of samples; either consecutive sample changer and/or SEC-SAXS (size-exclusion chromatography small-angle X-ray scattering) experiments, including column equilibration were also implemented. Automatic data processing and analysis are now managed via *Dahu*, an online server with upstream data reduction, data scaling and azimuthal integration built around *PyFAI* (*Python Fast Azimuthal Integration*), and data analysis performed using the open source *FreeSAS*. The results of this automated data analysis pipeline are displayed in *ISPyB/ExiSAXS*. The upgraded BM29 has been in operation since the post-EBS restart in September 2020, and here a full description of its new hardware and software characteristics together with examples of data obtained are provided.

## Abstract 2

As part of its Extremely Brilliant Source (EBS) upgrade project, the ESRF’s BioSAXS BM29 beamline was subject to a significant upgrade. Its bending magnet source was replaced by a two-pole wiggler, and a larger hybrid-pixel detector, a new sample changer and improved sample exposure cells were also installed and commissioned. Additionally, nearly all beamline software, including that for experiment control and data analysis, was renewed. Here the current status of the beamline is described, including new opportunities post-ESRF–EBS.

## 1. Introduction

To complement and supplement its highly successful facilities for macromolecular crystallography (MX) (Mueller-Dieckmann *et al.*, 2015 ▸), the ESRF has, since 2008, operated a small-angle X-ray scattering (SAXS) beamline dedicated to the study of the structures of biological macromolecules in solution. Originally located on ID14, where it shared a source with the ‘Quadriga’ complex of MX beamlines (Wakatsuki *et al.*, 1998 ▸), the beamline was moved to a dedicated ESRF bending magnet port (BM29) as part of the ESRF Phase I Upgrade Project (Theveneau *et al.*, 2013 ▸). This new beamline, BM29 (Pernot *et al.*, 2013 ▸), has been operational since June 2012.

BioSAXS at BM29 is highly automated and offers two main experimental modes: BioSAXS via a robotic sample changer (SC); and exploiting online size-exclusion chromatography (SEC), which purifies samples just prior to X-ray exposure (Brennich *et al.*, 2017 ▸; Tully *et al.*, 2021 ▸). Both modes are based around a common sample environment that enables switching between them with a simple software command that changes the position of a valve. In SC mode, operating at the maximal throughput, up to 500 measurements can be collected per day. The measurement throughput for SEC-SAXS is lower, limited by the length of time taken for a dedicated chromatography column run. The operation of BM29 relies on a high level of software development for beamline control, automated data-acquisition software, real-time data display and automatic data processing (Pernot *et al.*, 2013 ▸), which allows experimental users with no, or very little, prior knowledge of synchrotron-based BioSAXS beamlines to benefit from automatic sample loading/unloading, data collection, processing (conversion of a 2D image to a normalized 1D X-ray scattering profile) and analyses in both SC and SEC-SAXS online modes. Users obtain a normalized 1D scattering profile containing the raw data and automatically processed data sets that include the size (radius of gyration, maximum dimension and volume), molecular weight and in the near future a rough envelope of each sample within a minute from when a sample is measured. The results are logged, stored and displayed in the modified *ISPyB/ExiSAXS* database (De Maria *et al.*, 2015 ▸) where they can be followed (*e.g.* during remote access or mail-in experiments) and also downloaded. This allows, where necessary, for data collection strategies to be optimized ‘on the fly’.

Although many of the beamline’s instrumentation and software developments have been directed and produced in-house, BM29 also benefits from international collaborations. The automation of sample loading through the SC is the result of a collaboration between the EMBL (Grenoble and Hamburg outstations) and the ESRF. The results of automated data analysis pipeline and beamline metadata are displayed in *ISPyB/ExiSAXS*, an international collaboration between several synchrotrons and industry; the latest version uses an open source data reduction pipeline, *FreeSAS*, developed by the ESRF and other SAXS experts across Europe and the USA (Kieffer *et al.*, 2022 ▸).

The above approach has provided for a very successful, productive beamline providing a state-of-the-art resource for BioSAXS experiments with, to date, data from the beamline being cited in over 700 peer-reviewed publications (EPN-library, 2022 ▸). However, the beamline reopened in 2012 suffered from some limitations. The surface area of its in-air Pilatus 1M detector restricted both the accessible *q*-range and signal-to-noise ratios, while a relatively large beam size FWHM (100 µm × 500 µm, H × V) at the sample position was sub-optimal for experiments with small sample volumes. Moreover, BM29’s sample environment was rather inflexible and unsuited to experiments based on new technology (*i.e.* microfluidics) and to concurrent biophysical measurements (*i.e.* UV absorption measurements, *etc.*), which could provide added value to SAXS measurements. The recent (December 2018–August 2020) closure of the ESRF for its Extremely Brilliant Source (EBS) upgrade (ESRF, 2022*a* ▸) was therefore leveraged to provide an opportunity, carried out in collaboration with EMBL Grenoble and EMBL Hamburg, to upgrade BM29. Commensurate with the magnetic lattice of the ESRF–EBS storage ring lattice, the beamline’s bending magnet X-ray source was replaced by a two-pole wiggler, increasing the brilliance of BM29’s X-rays by a factor of ∼60. This was coupled with the refurbishment of almost the entire beamline sample environment and renewal/refactoring of beamline control and data reduction and analysis software.

## 2. New X-ray source and experimental set-up

**Figure 1:**
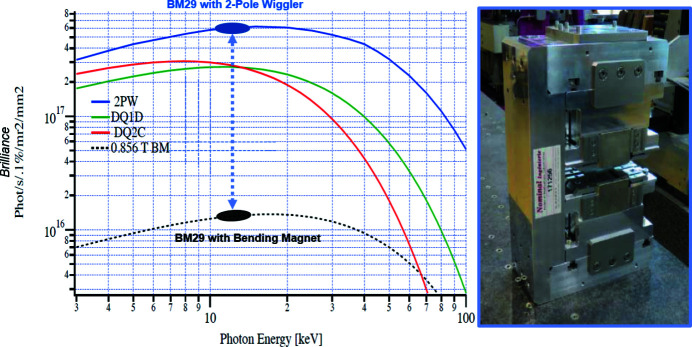
Increase in brilliance between BM29’s original bending magnet and current two-pole wiggler sources (photograph shown in inset). DQ2C and DQ1D correspond to combined dipole–quadrupole magnets located upstream and downstream, respectively, of the 2PW source in the ESRF–EBS lattice.

Due to the characteristics of the ESRF–EBS storage ring, the bending magnet source of BM29 was replaced by a two-pole wiggler (2PW) in March 2020. While this device produces a similar spectrum, has the same 0.86 T peak field and smooth traverse photon flux profile as BM29’s original bending magnet source, the 2PW has a much smaller source size, producing a theoretical increase by a factor of 60 in brilliance (Fig. 1 ▸). The main contributions to this increase derive from a smaller horizontal size and divergence of the electron beam in the centre of the new source (RMS): from 112 µm (BM) to 21 µm (2PW) and from 98.5 µrad (BM) to 23 µrad (2PW), respectively. The vertical source size also decreased from 11 µm (BM) to 4 µm (2PW), while vertical divergence increased from 0.4 µrad (BM) to 3 µrad (2PW). However, this increase does not lead to reduction in brilliance as the vertical divergence of the photon beam is dominated by the ‘natural’ divergence of single electron emission. Another feature of the two-pole structure is a horizontal offset of the electron beam of ±100 µm depending on the polarity of the first dipole. Two possible configurations are available: A and B corresponding to the wiggler fan (1.6 mrad) axis centred at −7.05 mrad and −8.65 mrad, respectively. As the original bending magnet axis of BM29 was at −9 mrad, the B configuration was adopted. This, however, required a realignment of all BM29 optical elements (Pernot *et al.*, 2013 ▸) to the new beam location in the horizontal plane by ∼2 cm at the front-end and ∼1 cm at the detector position. Due to the different location of the source in the ESRF–EBS lattice, the distance of the 2PW central point to the front-end increased from 22.54 m to 25.51 m with respect to the bending magnet source. Considering the resulting increased sample-to-source distance of 3 m which makes focusing at the detector plane less efficient (toroid mirror with fixed curvature and position), the installation of the 2PW source has resulted in a true gain in flux density of a factor of ten with respect to the previous source characteristics (and not 60 as expected from the brilliance increase). The beam size FWHM at the sample position is now ∼200 µm × 100 µm (H × V) with the photon flux increased to 1.4 × 10^13^ photons s^−1^ at an energy of 12.5 keV and 200 mA storage ring current. The photon flux was measured using a calibrated Si-diode placed at the sample position.

## 3. Sample environment

**Figure 2:**
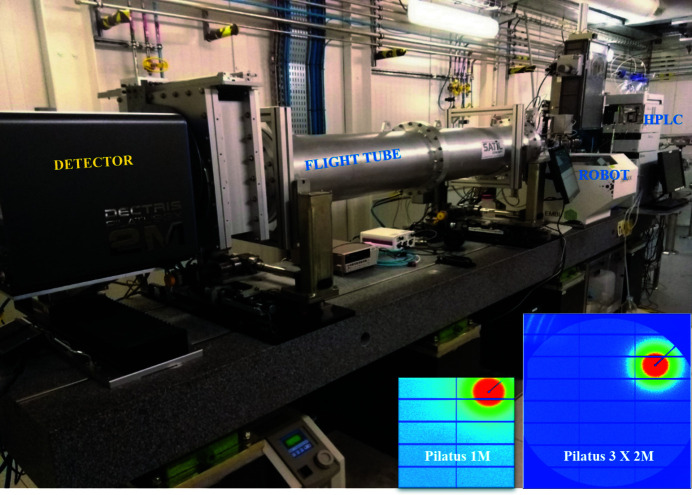
Experimental hutch of BM29 with new large-area detector Pilatus3 X 2M. Inset: comparison of images from Pilatus 1M and Pilatus3 X 2M.

**Figure 3:**
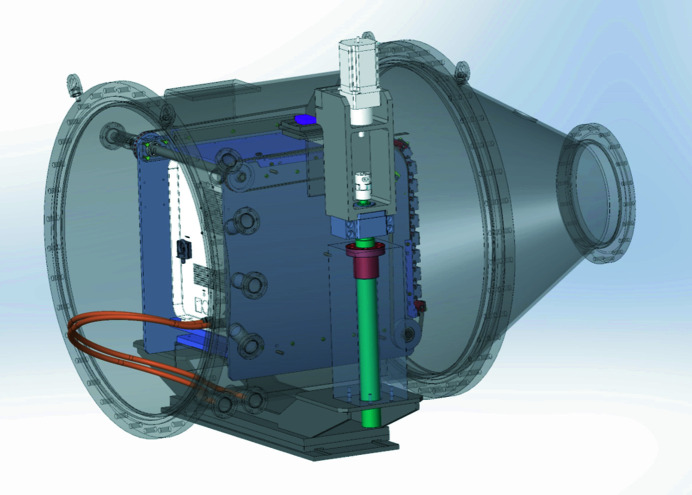
CAD design of the new flight tube end housing detector, ‘guillotine’ and beamstop with incorporated diode in vacuum.

As noted above, a shortcoming of the original BM29 was the surface area of its Pilatus 1M detector, which limited both the accessible *q*-range and signal-to-noise ratios, the latter exacerbated by the fact that the detector was installed in-air. In order to improve these limitations, a vacuum-compatible Pilatus3 X 2M detector (Dectris, Switzerland) with a sensitive area of 253.7 mm × 288 mm and frame rates of up to 250 Hz was installed and commissioned (Fig. 2 ▸). A new vacuum housing and flight tube end with incorporated ‘guillotine’, beamstop and diode is expected to be commissioned by the beginning of 2023 (Fig. 3 ▸). When this is available the *q*-range accessible at BM29 will increase further to 0.55–0.6 Å^−1^ (currently limited by the present flight tube diameter) and the reduced background when operating the detector *in vacuo* will improve the signal-to-noise ratio. This will enable lower concentration samples to be measured or decrease the collection time to reduce radiation damage.

**Figure 4:**
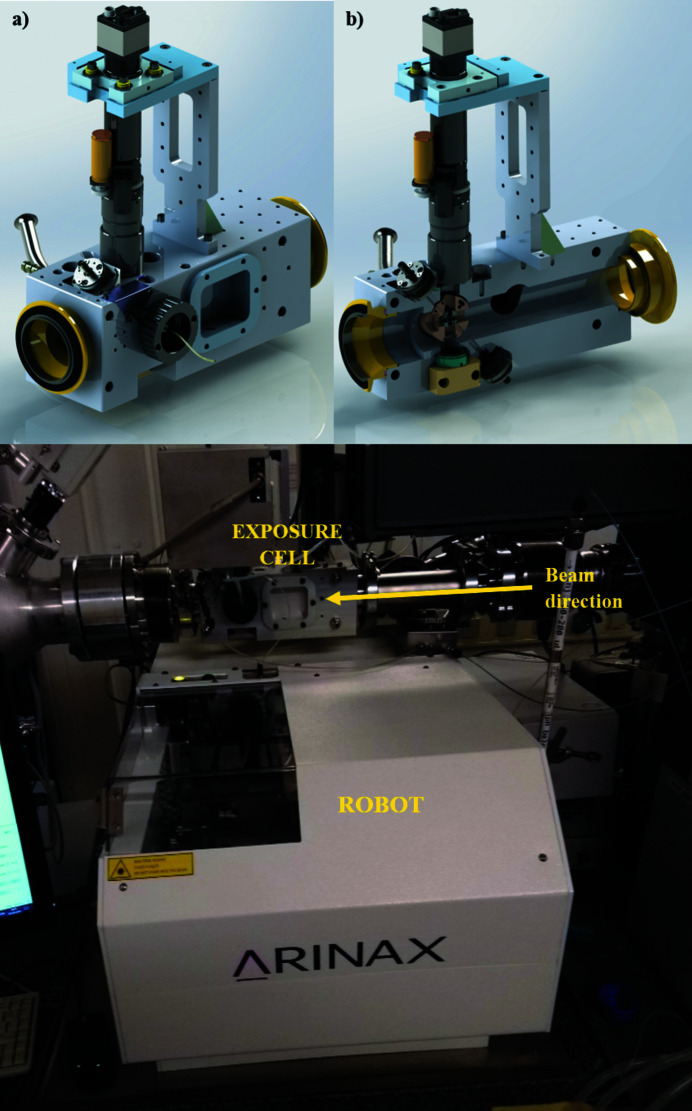
(Top) CAD design of the new SEU2A exposure (*a*) and a cut-through showing the position of the capillary and ports for spectrographic inserts (*b*). (Bottom) The SEU2A exposure cell *in situ* with new sample-changer robot.

As already stated, BioSAXS at BM29 is highly automated and offers two main experimental modes: BioSAXS via a robotic sample changer (SC) (Round *et al.*, 2015 ▸); and exploiting online size-exclusion chromatography (SEC), which purifies samples just prior to X-ray exposure (Brennich *et al.*, 2017 ▸; Tully *et al.*, 2021 ▸). During the ESRF–EBS shutdown and after ten years of service, the SC unit that was originally deployed on BM29 was replaced with an upgraded version (Fig. 4 ▸). A major advantage of the newly installed SC (Arinax) is that it is lower in height, allowing more space for newer and non-standard sample exposure units (SEUs), such as those developed in a collaboration between ESRF and EMBL (Figs. 4 ▸ and 5 ▸) described below. The newly deployed SC and dedicated SEU are optimized for smaller-diameter capillaries (1.0 mm versus 1.8 mm), which produce a faster and more laminar flow operating at a typical flow rate of 5 µl s^−1^ that should help mitigate and/or minimize radiation damage (Schroer *et al.*, 2018 ▸). Additionally, a more precise syringe for sample loading optimizes the loading of smaller sample volumes, as low as 5 µl.

Two SEUs, SEU2A and SEU2B, were developed for use with the upgraded SC unit. The first, SEU2A, is designed for use in ‘standard’ SC or SEC-SAXS experiments exploiting a capillary for optimal sample flow that has been future-proofed, with ports fitted for ultraviolet (UV) or dynamic light scattering (DLS) probes for any future complementary measurements and with a 20° opening angle design implemented, making it wide-angle X-ray scattering (WAXS) compatible (Fig. 4 ▸).

**Figure 5:**
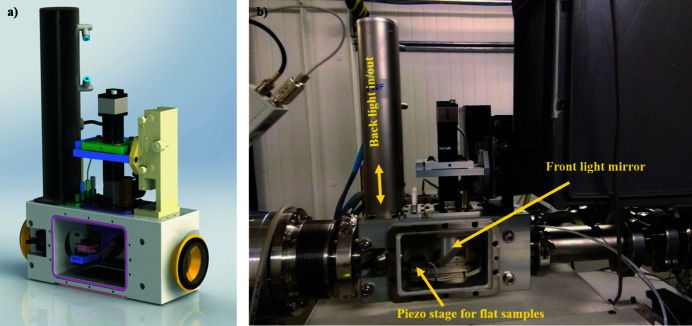
CAD design of the microfluidics-compatible SEU2B exposure cell (*a*) and SEU2B *in situ* (*b*).

The second, SEU (Fig. 5 ▸) is designed to be microfluidics compatible with feed-through connectors to allow sample flow and mixing. In-vacuum *x*/*y*/*z* piezo stages allow the scanning/rastering of a flat sample holder (microfluidic device or any 2D-compatible sample support holding other types of biological specimens, *e.g.* tissue) for sample centring and a retractable back light and front light sample illumination can help users optimize their experimental setup. The device is still in its commissioning phase and its software integration will be available from Spring 2023. In conjunction with the increased flux density available with the new 2PW source, this SEU2B will facilitate new BioSAXS investigations, such as using very small sample volumes (>5 µl) for time-resolved gel formation or mixing experiment.

The smaller, more intense, beam now available at the sample position brings the need for a more optimized and efficient beam ‘cleaning’ system. The current consecutive scatterless slits system [homemade hybrid metal-single-crystal (Si) slit, described by Li *et al.* (2008 ▸)] has been complemented with a motorized pinhole upstream to reduce parasitic scattering from optical elements. This also allows for quick changes (by moving the pinhole IN/OUT when suitable) in beam size FWHM decreasing from 200 µm × 100 µm (H × V) down to 50 µm × 50 µm. This new feature will aid in the development and integration of microfluidics devices with narrow channels (less than 100 µm), enabling future experimental setups with smaller X-ray compatible windows to be used without introducing strong parasitic scattering from channel/well walls.

## 4. Software developments

In order for the original BM29 to maintain a high level of automation while, at the same time, being accessible to non-expert users, the beamline featured the *BSXCuBE* (*BioSAXS Customized Beamline Environment*) software for experimental control and display, in real time, of both two-dimensional scattering images and integrated one-dimensional scattering curves (Pernot *et al.*, 2013 ▸). Further downstream processing was performed within the *EDNA* framework (Incardona *et al.*, 2009 ▸) and results displayed in *ISPyB/ExiSAXS* (De Maria Antolinos *et al.*, 2015 ▸; https://exi.esrf.fr/saxs), from where they can also be downloaded by the user. *BSXCuBE* morphed into *BSXCuBE2* with additional features, integrating the control of SEC-SAXS data acquisition and interacting with data analysis pipelines that encompassed the *ATSAS* package (Franke *et al.*, 2017 ▸). These iterations, developed under ESRF *Bliss Framework 4*, were powerful and served the ESRF BioSAXS user community for many years. However, they were difficult to maintain and did not allow for straightforward remote access experiments. For these reasons, in addition to upgrading the low-level beamline control macro language from *SPEC* (Swislow, 1987–2022 ▸; Certified Scientific Software, https://www.certif.com/) to the ESRF-developed Python-based *BLISS* (*BeamLine Instrument Support Software*) (Guijarro *et al.*, 2020 ▸; https://www.esrf.fr/BLISS), the decision was taken to completely re-engineer *BSXCuBE* producing an updated beamline control module, *BSXCuBE3* (Oskarsson *et al.*, 2019 ▸). *BSXCuBE3* is based on a similar technology stack and application concept to *MXCuBE3* (*Macromolecular Crystallography Customized Beamline Environment version 3*), the web-based user interface (Mueller *et al.*, 2017 ▸) installed at synchrotron-based MX beamlines at the ESRF and elsewhere that is developed under the auspices of the *MXCuBE* collaboration (Oskarsson *et al.*, 2019 ▸; https://mxcube3.esrf.fr/). Importantly, the on-line data analysis software was modified, with the open source *FreeSAS* (Kieffer *et al.*, 2022 ▸; https://www.silx.org/doc/freesas/dev/) replacing the previously used *ATSAS* package (Konarev *et al.*, 2006 ▸).

### 4.1. The *BSXCuBE3* graphical user interface

**Figure 6:**
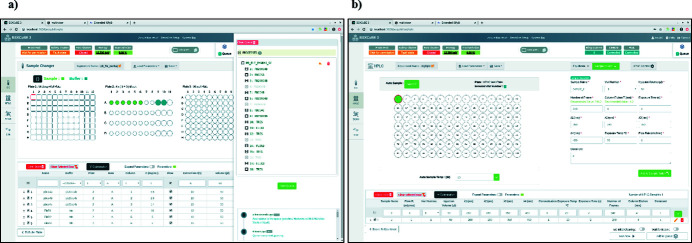
*BSXCuBE3* data acquisition modes. (*a*) Sample changer and (*b*) SEC-SAXS modes.

The layout and functionality of *BSXCuBE3* (Fig. 6 ▸) were developed with a ‘user first’ principal in mind, with the aim of producing an unobtrusive, intuitive beamline control interface accessible to new users but powerful enough for more expert users and beamline staff to carry out more complex experiments. Simple colours and an ergonomic stepwise progression with an emphasis on limiting the button clicks required to prepare and start an experimental data collection add to the simplicity of its use. The *MXCuBE3* framework on which *BSXCuBE* is broadly based consists of a back-end written in Python3 that is split into four parts: web server, control system integrator, common application logic and application-specific components. The reusable user interface components, many already existing in *MXCuBE3*, and a general-purpose back-end further facilitated good developmental practices by providing patterns and abstractions for both back-end and front-end development. This approach also allows faster development and easier maintenance. The front-end web client also shares the same key concepts as *MXCuBE3*, including beamline configuration and calibration, and incorporates an orthogonal view of the sample capillary. The logic behind the *BSXCuBE3* user interface is achieved by the components being handled by the ‘state management’ library *Redux* (https://redux-toolkit.js.org/) and implemented with *REACT* (https://reactjs.org) and *Bootstrap* (https://react-bootstrap.github.io) using *HTML5* and *ECMAScript 9* (https://github.com/tc39/ecma262). These advances in architecture and the design of new features in *BSXCuBE3* are intended to enhance the automation available on BioSAXS beamlines and facilitate the integration of new sample environments, such as microfluidics. The access to the application from any web browser natively allows for remote access for beamline control.

### 4.2. *FreeSAS* data analysis software

**Figure 7:**
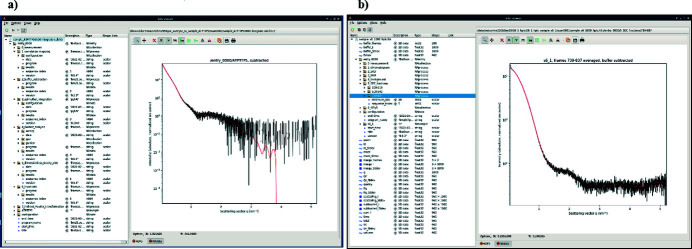
Data analysis results visualized by *silx* view. Subtracted curve in (*a*) sample changer and (*b*) SEC-SAXS modes.

An essential aspect of high-throughput BioSAXS experiments is feedback, in as close to real time as possible, regarding the quality of the scattering curves produced for any given sample. The automatic data processing and analysis pipelines available for experiments at BM29 provides this. Originally built using the *EDNA* framework (Brennich *et al.*, 2016 ▸), the BM29 data processing and analysis pipelines are now managed via *Dahu* (named after the mythical alpine goat), an online *JSON-RPC* data analysis server operated over *Tango* (https://www.silx.org/doc/dahu/dev/). Here, upstream data reduction (data scaling and azimuthal integration) is built around *PyFAI* (*Python fast azimuthal integration*) (Ashiotis *et al.*, 2015 ▸; Kieffer *et al.*, 2020 ▸). The BioSAXS data analysis performed using the open source *FreeSAS* (Kieffer *et al.*, 2022 ▸; https://www.silx.org/doc/freesas/dev/) includes curve averaging that accounts for radiation damage, background subtraction (matched buffer measured before and after each sample) and routine analysis on the resulting scattering curve carried out with *CorMap* (Franke *et al.*, 2015 ▸), *autorg* (Hopkins *et al.*, 2017 ▸) or *Auto_GPA* (Putnam, 2016 ▸), invariants (Porod volume, volume of correlation mass calculation; Rambo & Tainer, 2013 ▸) and *BIFT* (*Bayesian inverse Fourier transform*; Vestergaard & Hansen, 2006 ▸). All data analysis results are saved in hierarchical data format (HDF) that can be visualized with *silx* view for both SC and SEC-SAXS modes, respectively (Fig. 7 ▸). *Ab initio* reconstructions are not yet a part of the automated pipeline and for this the plan is to implement the open source software *DENSS* (*density from solution scattering*; Grant, 2018 ▸).

**Figure 8:**
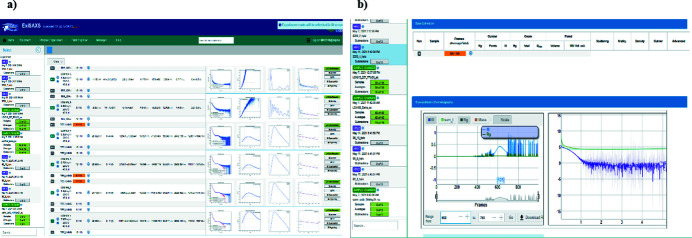
Results of (*a*) sample changer and (*b*) SEC-SAXS experiments displayed in *ISPyB/ExiSAXS.*

Results of the automated analysis are accessible in a modified *ISPyB/ExiSAXS* database for SAXS (De Maria Antolinos *et al.*, 2015 ▸). This allows for crosschecks for consistency between different runs, providing ‘on the fly’ feedback regarding the need for repeat or extra measurements to complete the current experiment. Examples of the *ISPyB/ExiSAXS* visualization for a batch SC and SEC-SAXS measurements are shown in Fig. 8 ▸.

## 5. Recent examples of data quality collected on BM29

**Figure 9:**
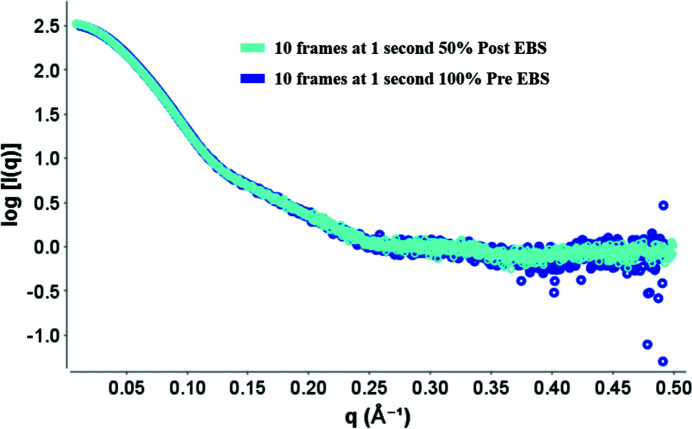
Intensity (*I*) versus scattering vector (*q*) for protein BSA pre- and post-ESRF–EBS. Each sample contained 5 mg ml^−1^ of BSA in 50 m*M* Hepes buffer pH 7.5. Dark blue: 100% transmission pre-EBS in a capillary of 1.8 mm diameter. Cyan: 50% transmission post-EBS in 1 mm capillary. Ten frames of 1 s each were averaged for each sample.

In principle, the collection strategy for BM29 has not changed since the ESRF–EBS upgrade. Generally, each measurement in SC mode is composed of ten frames with a 1 s exposure time per frame. The increased flux and decreased beam size at the sample position give a stronger signal with an increased signal-to-noise ratio as seen in Fig. 9 ▸, and no radiation damage is seen when compared with samples taken before the ESRF–EBS upgrade.

**Figure 10:**
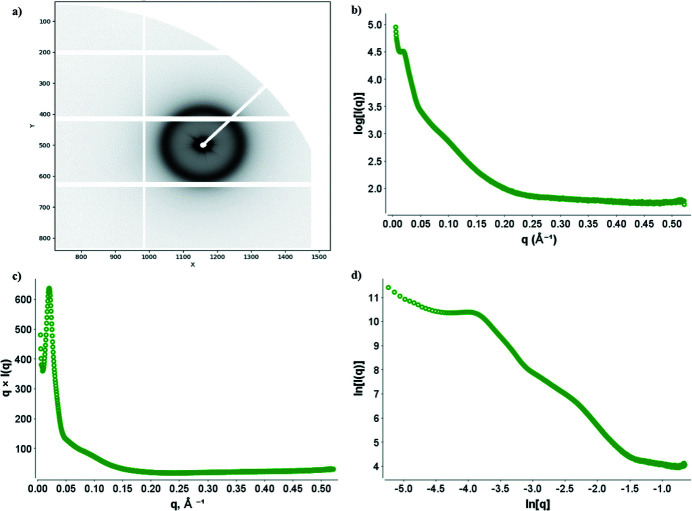
Scattering of gel forming protein, GFP-Elf3-Q0. (*a*) Image of the Pilatus3 X 2M detector showing halo of scattering intensity. (*b*) Intensity (*I*) versus scattering vector (*q*) for GFP-Elf3-Q0 showing a structure factor peak present. (*c*) By multiplying the *y*-axis by *q*, a total intensity plot is created emphasizing the structure factor peak. (*d*) A log-intensity plot; the rising Guinier region is indicative of a heterogenous species.

The smaller beam size post-ESRF–EBS coupled with a new sample environment unit (SEU2B microfluidics compatible) has enabled the use of 3D printed microfluidic devices. We tested the new possibility using the *Arabidopsis* plant protein *EARLY FLOWERING 3* (*ELF3*) that contains a prion-like domain with a variable-length polyglutamine tract, whose length varies across natural *Arabidopsis* accessions (Silva *et al.*, 2020 ▸). When introduced to physicochemical changes such as salt, pH, heat or increased concentration, the protein undergoes reversible liquid–liquid phase transition (Jung *et al.*, 2020 ▸). However, when heated beyond 27°C, the protein over time forms a stable gel. Using a 3D printed device mounted within the new SEU2B, in vacuum, the gel was sandwiched between Kapton tape and X-ray scattering was measured. Air was measured in the same device for background subtraction. The scattering profile (Fig. 10 ▸) showed a large structure factor peak formed at *q* = 0.021 Å^−1^ that corresponds to a *d*-spacing of ∼300 Å, indicating the intermolecular ordering within the gel. This experiment displays the ability of BM29 to expand its possible repertoire of sample measurement options offered post-ESRF–EBS from solution SAXS to gels and semi-solid materials.

## 6. Current status and future development

After the ESRF–EBS upgrade, the BM29 BioSAXS beamline has been in full user operation since mid-September 2020 and has hosted more than 216 experimental sessions of which 122 were mail-in experiments during the recent health crisis. Access to the experimental facilities is offered through the ESRF MX BAG (Block Allocation Group) and Rolling access system (ESRF, 2022*b* ▸). The latter, upon approval through ESRF’s external beam time allocation panel (BTAP), allows data collection within six weeks after the experimental proposal submission. Refer to Table 1 ▸ for beamline schematics post-EBS upgrade.

To promote the study of proteins and nucleic acids in solution with small-angle scattering (SAS) in Grenoble, France, a joint application process was implemented for both neutron small-angle scattering (SANS) and X-ray small-angle scattering experiments (SANSAXS BAG) in collaboration with the ILL, France (ESRF, 2022*c* ▸). This allows the complementary information provided by neutron scattering (*i.e.* contrast variation; Jacrot, 1976 ▸) and X-ray scattering to be obtained in a single visit to the EPN campus in Grenoble.

Future developments on the beamline will include the addition of a WAXS experimental setup. To allow this, the new BioSAXS sample exposure unit was designed with an increased opening angle of up to 20° to accommodate a new WAXS detector. This shifts the upper limit of the detectable *q*-range from 0.6 Å^−1^ to 2.5 Å^−1^ and renders the biologically relevant part of the WAXS regime accessible. For proteins and nucleic acids, the WAXS signal contains secondary structure information. For example, for nucleic acids, inter-strand pair distance correlations from major and minor groove spacing and helix radius result in sequence-specific peaks in the WAXS region (Makowski, 2010 ▸). For proteins, WAXS has been used to screen for ligand binding and characterization of fold. It is particularly powerful when combined with molecular dynamics simulations, where it allows testing of predictions on the sub-nanometre scale. The WAXS range hence provides a powerful tool for assessing models and predictions of protein and nucleic acid structures.

To make full use of the new sample environment, a suite of microfluidic devices will be developed and tested; once validated, these can be offered to BM29 users. This will encompass a range of different experiments from *in situ* mixing of buffers or ligands enabling time-resolved SAXS to the measurement of gels and fibre formation and high-throughput screening of samples and ligands.

*AlphaFold* (Jumper *et al.*, 2021 ▸) is an innovative and accessible tool to easily predict structures – however, highly flexible domains and low complexity regions are beyond reliable calculations; SAXS is still one of the most powerful techniques to measure these intrinsically difficult systems. Given these developments we expect an increase in user requests for BioSAXS, and BM29 post-ESRF–EBS is well placed and adapted for these demands.

## Figures and Tables

**Table 1:** BM29 beamline schematics pre- and post-EBS upgrade

	BM29 pre-EBS	BM29 post-EBS
Source type	Bending magnet centred at −9 mrad	Two-pole wiggler centred at −8.65 mrad
Mirrors	1.1 m-long Rh-coated toroid, 4 mrad	1.1 m-long Rh-coated toroid, 4 mrad
Monochromator	Double multilayer, Si substrate with Ru/B_4_C coating, spacing 2.96 nm	Double multilayer, Si substrate with Ru/B_4_C coating, spacing 2.96 nm
Energy range (keV)	7–15	7–15
Wavelength range (Å)	0.82–1.77	0.82–1.77
Beam size FWHM (focused, typical) (µm)	1000 × 500 at sample plane	200 × 100 at sample plane
Flux (collimated, typical) (photons s^−1^)	2 × 10^12^ (at 12.5 keV)	1.4 × 10^13^ (at 12.5 keV)
*q*-range (Å^−1^)	0.007–0.5	0.007–0.55
Sample environments	Robot, HPLC	Robot, HPLC and scanning cell
Experimental temperature range (°C)	4–60	4–60
Sample cell	Quartz capillary, 1.8 mm diameter	Quartz capillary, 1 mm diameter
Detector type	CMOS hybrid pixel	CMOS hybrid pixel
Detector model	Pilatus 1M	Pilatus3 X 2M (in vacuum from 2023)
Detector area (mm)	169 × 180	253.7 × 288
2θ capabilities	0–4°	0–4°
Sample-to-detector distance (m)	2.81	2.81 (2.74 when detector in vacuum)
